# The Dynamic Relationship Between Cortical Oxygenation and End-Tidal *CO*_2_ Transient Changes Is Impaired in Mild Cognitive Impairment Patients

**DOI:** 10.3389/fphys.2021.772456

**Published:** 2021-12-09

**Authors:** Vasilis Z. Marmarelis, Dae C. Shin, Rong Zhang

**Affiliations:** ^1^Biomedical Engineering Department, University of Southern California, Los Angeles, CA, United States; ^2^Institute for Exercise and Environmental Medicine, UT Southwestern Medical Center, Dallas, TX, United States

**Keywords:** cortical tissue oxygenation, cerebral *CO*_2_ vasoreactivity, cerebral blood flow regulation, regulation of cerebral perfusion, cerebrovascular regulation, dynamic vasomotor reactivity, mild cognitive impairment

## Abstract

**Background:** Recent studies have utilized data-based dynamic modeling to establish strong association between dysregulation of cerebral perfusion and Mild Cognitive Impairment (MCI), expressed in terms of impaired CO_2_ dynamic vasomotor reactivity in the cerebral vasculature. This raises the question of whether this is due to dysregulation of central mechanisms (baroreflex and chemoreflex) or mechanisms of cortical tissue oxygenation (CTO) in MCI patients. We seek to answer this question using data-based input-output predictive dynamic models.

**Objective:** To use subject-specific data-based multivariate input-output dynamic models to quantify the effects of systemic hemodynamic and blood CO_2_ changes upon CTO and to examine possible differences in CTO regulation in MCI patients versus age-matched controls, after the dynamic effects of central regulatory mechanisms have been accounted for by using cerebral flow measurements as another input.

**Methods:** The employed model-based approach utilized the general dynamic modeling methodology of Laguerre expansions of kernels to analyze spontaneous time-series data in order to quantify the dynamic effects upon CTO (an index of relative capillary hemoglobin saturation distribution measured *via* near-infrared spectroscopy) of contemporaneous changes in end-tidal CO_2_ (proxy for arterial CO_2_), arterial blood pressure and cerebral blood flow velocity in the middle cerebral arteries (measured *via* transcranial Doppler). Model-based indices (physio-markers) were computed for these distinct dynamic relationships.

**Results:** The obtained model-based indices revealed significant statistical differences of CO_2_ dynamic vasomotor reactivity in cortical tissue, combined with “perfusivity” that quantifies the dynamic relationship between flow velocity in cerebral arteries and CTO in MCI patients versus age-matched controls (*p* = 0.006). Significant difference between MCI patients and age-matched controls was also found in the respective model-prediction accuracy (*p* = 0.0001). Combination of these model-based indices *via* the Fisher Discriminant achieved even smaller *p*-value (*p* = 5 × 10^–5^) when comparing MCI patients with controls. The differences in dynamics of CTO in MCI patients are in lower frequencies (<0.05 Hz), suggesting impairment in endocrine/metabolic (rather than neural) mechanisms.

**Conclusion:** The presented model-based approach elucidates the multivariate dynamic connectivity in the regulation of cerebral perfusion and yields model-based indices that may serve as physio-markers of possible dysregulation of CTO during transient CO_2_ changes in MCI patients.

## Introduction

One of the most important mechanisms for the regulation of cerebral perfusion pertains to *CO*_2_ vasomotor reactivity, whereby an increase in blood *CO*_2_ tension causes a decrease of cerebrovascular impedance (through vasodilation) and, consequently, an increase of cerebral blood flow. The latter enables the gradual reduction of the original surge in blood *CO*_2_ tension in order to achieve homeostasis (from hypercapnia to normocapnia). This mechanism also acts in the reverse manner in the event of a decrease in blood *CO*_2_ tension to achieve normocapnia from a state of hypocapnia (Maeda et al., 1993; Ainslie and Duffin, 2009; Ainslie and Tzeng, 2010; Willie et al., 2012, 2014; Carter et al., 2016; Hoiland and Ainslie, 2016; Caldwell et al., 2021). The vasomotor reactivity mechanism, which makes *CO*_2_ a potent vasodilator, was also revealed experimentally through magnetic resonance imaging studies (Cloverdale et al., 2014; Verbree et al., 2014). If this critical mechanism fails, then the ensuing conditions of prolonged hypercapnia or hypocapnia in cerebral tissue may have detrimental effects on the proper function of cerebral cells, including glial cells involved in neurovascular coupling that support proper neuronal and cognitive function. This view is supported by several studies of cerebral microcirculation and neurodegenerative disease, with particular focus on Mild Cognitive Impairment (MCI) and Alzheimer’s Disease (AD) (Franceschi et al., 1995; Terbor et al., 2000; de la Torre, 2002, 2004; Iadecola and Gorelick, 2003; Hachinski and Iadecola, 2004; Iadecola, 2004, 2013; Tong et al., 2005; Silvestrini et al., 2006; Claassen et al., 2009; Nicolakakis and Hamel, 2011; Tan and Taylor, 2014; Alwatban et al., 2019).

In our previous work, we quantified (through a model-based approach utilizing spontaneous hemodynamic time-series data) the *CO*_2_ dynamic vasomotor reactivity (DVR) of cerebral vasculature and the cortical oxygenation response (COR) to changes in blood *CO*_2_ tension, discovering significant impairment of both DVR and COR in patients with amnestic MCI relative to age-matched controls (Marmarelis et al., 2017). These findings are consistent with the results of previous studies that have reported a correlation between cognitive impairment and deficits in *static* cerebral *CO*_2_ vasoreactivity measured through *CO*_2_ inhalation or breath-holding (Maeda et al., 1993; Franceschi et al., 1995; Terbor et al., 2000; de la Torre, 2002, 2004; Iadecola and Gorelick, 2003; Hachinski and Iadecola, 2004; Iadecola, 2004; Tong et al., 2005; Silvestrini et al., 2006). Notable is a large retrospective study concluding that dysregulation of cerebral perfusion is the earliest and strongest of all known and examined risk factors in the pathogenesis of Alzheimer’s Disease (AD) (Iturria-Medina et al., 2016).

In this broad effort, our work has sought to contribute quantitative measures of cerebral flow regulation (and possible dysfunction) in the form of data-based DVR indices computed from predictive dynamic models of how beat-to-beat cerebral flow velocity or cortical tissue oxygenation (CTO) are influenced by changes in arterial blood pressure and end-tidal *CO*_2_ (a surrogate for arterial blood *CO*_2_ tension) under resting spontaneous conditions (Marmarelis et al., 2012, 2013, 2014, 2016, 2017, 2019). The model-based DVR index may serve as a useful “physio-marker” of *CO*_2_-mediated regulation of cerebral circulation in cerebrovascular disease and/or dysregulation of cerebral perfusion, possibly associated with cognitive impairment. This approach was also extended to the study of the heart-rate chemoreflex of MCI patients (vs age-matched control subjects) and significant impairment in the chemoreflex gain was found in MCI patients that correlated strongly with their respective deficit in DVR at middle cerebral arteries—but not with the deficit in cortical tissue oxygenation (CTO) at the lateral prefrontal cortex (Marmarelis et al., 2020). This intriguing finding led us to explore how much of the CTO deficit depends on factors other than chemoreflex-dependent *CO*_2_ regulatory action upon blood flow in large cerebral arteries. It is important to clarify that the term “CTO” (used by the manufacturer of the near infrared spectroscopy device that makes these measurements) is actually an index of relative hemoglobin saturation distribution in the arterial/venous compartments. This relative distribution can be altered by capillary recruitment caused by changes in cerebral blood flow, thus providing a “directional hypothesis” to the present study.

To this purpose, we include in this study the measurements of blood flow velocity at the middle cerebral arteries as another “input” of our predictive dynamic model (in addition to the inputs of arterial blood pressure and end-tidal *CO*_2_) with output being the CTO measurements at the lateral prefrontal cortex. This data-based model allows the quantification of the dynamic effects of transient systemic *CO*_2_ changes upon localized CTO at the lateral prefrontal cortex—separately from blood *CO*_2_ effects on cerebral arteries (vasomotor reactivity). This may prove useful for quantifying CTO regulation/dysregulation in cerebrovascular or neurological disease at the level of small/micro cortical vessels. Furthermore, proper scientific interpretation of the obtained predictive models may provide valuable insights into the physiological mechanisms that affect the regulation of cortical perfusion and oxygenation in healthy subjects and patients.

To provide some additional insight into the fundamental rationale of our modeling approach, we note that input-output models of dynamic systems seek to describe how the present value of the output signal is constructed from the epochs of the input signals—an epoch being defined as the input signal segment that ends at the present time and reaches back into the past over a finite extent termed the “memory” of the system with respect to a given input-output relation. For a general nonlinear dynamic system/model, a universally valid description is offered by the Volterra functional series and its variants that have the form of multiple convolution integrals (Marmarelis, 2004). The estimation of such a general nonlinear dynamic model from input-output data is a challenging task, albeit feasible under certain conditions (Marmarelis, 2004; Marmarelis et al., 2012, 2013, 2016). This task becomes much simpler and more robust when the model seeks to capture only the *linear* dynamics of the system. This is the case of this study for which the employed three-input linear dynamic model has the form of the sum of three convolutions (one for each input) as indicated in Equation (1). A complete characterization of the linear dynamics of this system is achieved when the three “kernels” of the convolutional terms in Equation (1) are estimated from input-output data. These kernels are characteristic of each system and can be viewed as “the patterns of weighing the input epochs in order to construct the present output value.” The kernels can be estimated robustly when the Laguerre expansion technique is used (Marmarelis, 2004), as outlined in Appendix.

The present paper employs this quantitative methodology to extract predictive models of the dynamic regulation of cortical oxygenation (viewed as an “output” variable) as a function of concurrent spontaneous changes of blood *CO*_2_ tension and the key hemodynamic variables of arterial blood pressure and cerebral blood flow in the middle cerebral arteries (viewed as three “inputs”). The obtained subject-specific models offer novel insights into the dynamics of this system and can advance our scientific understanding when properly interpreted. They can also generate model-based “physio-markers” regarding various aspects of the regulation of cerebral perfusion or oxygenation and its possible association with MCI. For example, the findings indicate significant reduction in the CTO response of MCI patients, relative to age-matched controls, in response to a step change in blood *CO*_2_. This statistical differentiation increases when the CTO response to a step change in cerebral blood flow is also taken into account. The full potential scientific and diagnostic utility of this approach remains an important goal for future studies as more relevant knowledge is accumulated.

## Materials and Methods

### Data Collection

We collected the following time-series datasets over 5 min in 36 patients with amnestic MCI and 12 age-matched cognitively normal controls:

(1) mean arterial blood pressure (ABP) over each heart-beat, measured continuously with finger photo-plethysmography (Finapres); the heart-beat interval was also determined from Finapres;
(2) end-tidal *CO*_2_ (CO_2_) measured *via* nasal cannula using capnography (Criticare Systems);
(3) mean cerebral blood flow velocity (CFV) over each heart-beat, measured continuously in the middle cerebral arteries using a transcranial Doppler (TCD) probe (Multiflow, DWL) placed over the temporal window and fixed at constant angle with a custom-made holder;
(4) cortical tissue oxygenation (CTO) as tissue oxygenation index (TOI), defined as the ratio of oxyhemoglobin to total hemoglobin multiplied by 100, measured *via* Near Infrared Spectroscopy (NIRS) (Hamamatsu). As noted in Introduction, this index is not quantifying directly “tissue oxygenation” but rather the relative hemoglobin saturation distribution in the arterial/venous compartments (which we assume to be related to tissue oxygenation).

All patients/subjects participated voluntarily in this study and signed the Informed Consent Form that has been approved by the Institutional Review Board of the UT Southwestern Medical Center and Texas Health Presbyterian Hospital Dallas. Participants were recruited from the University of Texas Southwestern Medical Center Alzheimer’s Disease Center, senior centers in the Dallas-Fort Worth metropolitan area, and local newspaper advertisements. The diagnosis of amnestic MCI was based on modified Petersen criteria (Petersen et al., 2001). The scores of the Mini-Mental State Exam (MMSE) were used to assess global cognitive function. The scores of the Delayed Logical Memory Recall (DLMR) subtest of the Wechsler Memory Scale-Revised were used to assess objective memory loss in patients with MCI, as recommended by the ADNI project.^1^ Healthy older adults (control subjects) were cognitively normal and did not have MCI or dementia. Both healthy controls and patients with MCI were excluded if they had major neurologic, vascular, or psychiatric diseases, uncontrolled hypertension, clinically diagnosed or self-reported diabetes mellitus, clinical histories of stroke, unstable heart diseases, sleep apnea, body mass index (BMI) ≥ 35 kg/m^2^, and current or past history of smoking. Participants were instructed to abstain from caffeinated beverages, alcohol, and vigorous exercise over the 24 h prior to the clinical visit. All hemodynamic data were collected in a quiet, environmentally controlled laboratory with an ambient temperature of 22 degrees Celsius, under resting seated conditions.

All measurements were non-invasive, safe and comfortable for the subjects. After 20 min of rest, 5-min recordings were made at an initial sampling rate of 1 KHz and subsequently pre-processed prior to analysis as described below. The gender composition, the age (mean and standard deviation) and the neuropsychological scores (MMSE and DLMR) of the patients and controls are given in Table 1.

**TABLE 1:** Cohort demographics and Mean (SD) of neuropsych scores and time-series data averages.

Subjects	Gender	Age	MMSE score	DLMR score	TOI^‡^ in %	CFV^§^ in cm/sec	CO_2_^¶^ in mmHg	ABP^#^ in mmHg
12 Controls	7 male and 5 female	68.80 (5.14)	29.00 (0.95)	14.17 (1.85)	68.01 (7.15)	49.63 (13.94)	35.10 (3.33)	91.95 (14.85)
36 Patients	12 male and 24 female	66.19 (6.25)	29.31 (0.83)	8.86 (2.31)	63.17 (7.97)	47.01 (10.91)	35.88 (2.54)	91.44 (13.23)
***p*-value**	*0.12* *	*0.16*	*0.32*	**3.3 × 10^–8^**	*0.06*	*0.56*	*0.46*	*0.91*

*^‡^Tissue Oxygenation Index (measured via Near Infrared Spectroscopy at the lateral prefrontal cortex).*

*^§^ Cerebral Blood Flow Velocity (measured via Transcranial Doppler at the middle cerebral arteries).*

*^¶^ End-tidal CO_2_ (proxy for arterial CO_2_ tension, measured via capnography through nasal cannula).*

*^#^Arterial Blood Pressure (intra-beat average measured via photo-plethysmography at the finger).*

*Computation of this p-value used +1 for each female and 0 for each male subject/patient.

*Bold values represents statistically significant.*

### Data Pre-processing

**FIGURE 1:**
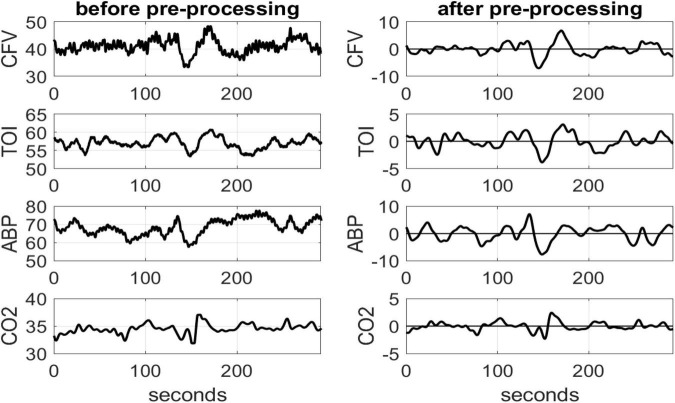
Illustrative time-series data over 5 min for one control subject, representing spontaneous variations of CFV (top panel), TOI (2nd), ABP (3rd) and CO_2_ (bottom panel), before (left column) and after (right column) pre-processing that was described in the text. The units are: cm/sec for CFV, % for TOI and mmHg for ABP and CO_2_.

The collected highly sampled data of ABP, CFV and TOI were reduced to beat-to-beat time-series data using averages over the respective heart-beat (unevenly sampled) and were re-sampled every 0.25 s *via* cubic-spline interpolation. Breath-to-breath end-tidal *CO*_2_ values were placed at the mid-point of each breath (unevenly sampled) and were re-sampled every 0.25 s *via* cubic-spline interpolation to make the data-samples of all physiological variables contemporaneous. All time-series data were high-pass filtered (*via* subtraction of a 2-min moving-average Hanning window) to remove the constant baseline and very low frequency content below 0.01 Hz, and low-pass filtered using an 8-s Hanning window in order to alleviate the effects of respiratory sinus arrhythmia and concentrate on dynamic effects below 0.2 Hz where hemodynamic regulation is thought to take place (Marmarelis et al., 2020). Figure 1 shows illustrative time-series data (raw and Pre-processed) for a control subject over 5 min.

### Modeling Methodology

In order to obtain the requisite predictive dynamic models, we employ the input-output modeling methodology that has been pioneered by our lab (Marmarelis, 2004; Marmarelis et al., 2012, 2013, 2014, 2016, 2017, 2019) and outlined briefly below and in Appendix. This method has been extensively tested and validated over the last 30 years in numerous published studies of various physiological systems, with the majority of such studies being on neural systems. In this study, data-based estimates of input-output predictive models are obtained under resting conditions that describe the dynamic relationships between three putative “inputs”: (1) beat-to-beat data of mean arterial blood pressure (ABP) measured *via* finger photo-plethysmography; (2) beat-to-beat data of mean cerebral flow velocity (CFV) measured *via* transcranial Doppler at the middle cerebral arteries; (3) breath-to-breath data of end-tidal *CO*_2_ (CO_2_) measured *via* capnography through a nasal cannula; and the putative “output” of beat-to-beat data of mean CTO measured *via* Near Infrared Spectroscopy as Tissue Oxygenation Index (TOI) at the lateral prefrontal cortex. The estimated model for each of 36 MCI patients (MP) and 12 age-matched control subjects (Controls) is then used to compute indices quantifying certain physiological functions of interest *via* the proper simulations. For instance, a model-based index of CTO/TOI response to CFV is computed as the time-average over 50 s of the model-predicted TOI response to a unit-step (1 cm/s) change of CFV, as described later. These input-output predictive dynamic models are estimated using the novel methodology of Laguerre kernel expansions and Principal Dynamic Modes that was pioneered by our lab and has been shown to yield accurate model estimates from noisy and relatively short datasets (Marmarelis, 2004). The key to the advocated modeling approach is the *general input-output predictive model form for all linear systems* that has the convolutional form shown below for the model of the dynamic relationship between the chosen three inputs (ABP, CO_2_, CFV) and the output (CTO/TOI):


(1) $$\begin{array}{c} y\left(n\right)=k_{0}+\sum_{m=0}^{M_{p}}k_{p}\left(m\right)p\left(n-m\right)+\sum_{m=0}^{M_{x}}k_{x}\left(m\right)x\left(n-m\right)\\ \;+\sum_{m=0}^{M_{z}}k_{z}\left(m\right)z\left(n-m\right) \end{array}$$

where: *y(n)* denotes the *n-th* CTO/TOI output sample; the discrete time-series data *p(n)*, *x(n)* and *z(n)* denote the three inputs ABP, CO_2_ and CFV respectively; while *k*_*p*_, *k*_*x*_ and *k*_*z*_ denote the three “kernels” of the model with respect to the inputs *p(t), x(t)*, and *z(t)*, respectively. The summations represent discrete convolution operations, which constitute a general mathematical/computational model form for all *linear* dynamic systems with invariant characteristics over time. This general modeling approach has also been extended to the analysis of *nonlinear* dynamic systems using multi-dimensional convolutions (Marmarelis, 2004; Marmarelis et al., 2012, 2014)—although longer data-records are then required for the reliable estimation of this more complicated general nonlinear model form. The system under study has some nonlinear characteristics, but the linear model approximation is deemed adequate for our purposes, based on our previous studies, since robust kernel estimates can be obtained from the available data (relatively short and noisy). The three kernels of the model in Eq. (1) are the key entities that describe fully the dynamic characteristics of this *general linear* input-output model. The kernels allow prediction of the output *y(n)* for *any* given set of input waveforms *p(n), x(n)*, and *z(n)*. An outline of the kernel estimation procedure from the input-output time-series data is provided in Appendix.

## Results

**FIGURE 2:**
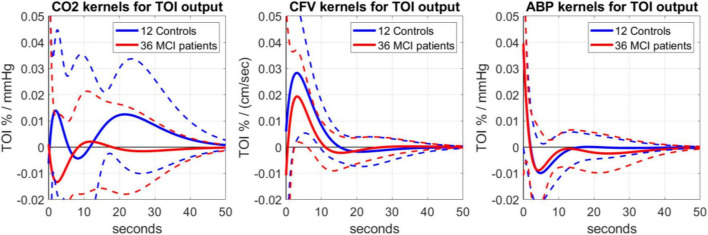
Average kernel estimates (± 1 SD bounds marked with dotted lines) over 12 Controls (blue line) and 36 MCI patients (red line), quantifying the dynamic effects upon TOI (output) of changes in the CO_2_ input (left), CFV input (middle) and ABP input (right).

Following the methodological procedures outlined above, we obtained data-based kernel estimates of the predictive dynamic models for the effects upon TOI of changes in ABP, CO_2_ and CFV. The average kernels and their standard deviation (SD) bounds are shown in Figure 2 for the 36 MCI patients (red line) and 12 Controls (blue line). The average kernel waveforms appear to be similar between MCI patients vs. Controls for the ABP and CFV inputs (see right and middle panels, respectively)—although the average CFV-input kernel has somewhat smaller magnitude for the MCI patients, indicating reduced blood flow conductivity from the middle cerebral arteries to the arterioles and capillaries of the prefrontal cortex. Importantly, the average kernel waveforms are distinctly different between MCI patients vs. Controls for the CO_2_ input (see left panel of Figure 2), with the MCI patients kernel exhibiting a negative early component (for lags < 8 s) and a smaller oscillatory component for longer lags, whereas the Controls kernel exhibits a large positive early component (for lags < 4 s) and a larger positive component for lags > 10 s that peaks around 20 s lag.

**FIGURE 3:**
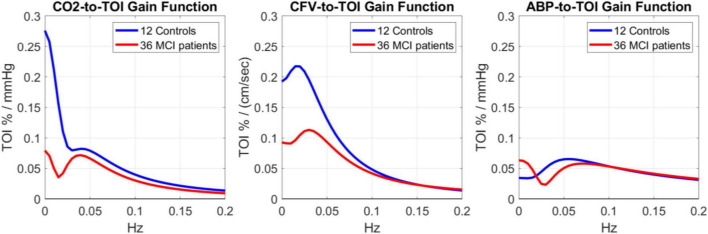
Gain Functions of the average kernel estimates shown in Figure 2 for 12 Controls (blue line) and 36 MCI patients (red line), which quantify the frequency-response characteristics of the TOI output to changes in CO_2_ input (left), CFV input (middle) and ABP input (right).

The three pairs of average kernels are also compared in the frequency-domain in Figure 3, using the magnitudes of the respective Discrete Fourier Transforms, which are the Gain Functions (GF) of the respective Transfer Functions. For the CO_2_ input (left panel of Figure 3), the GF appears different between Controls and MCI patients for frequencies < 0.04 Hz (by a factor of 4 on the average). The MCI patients exhibit a resonant peak around 0.04 Hz, while the Controls exhibit a strong low-pass (i.e., integrative) characteristic at frequencies < 0.04. These notable differences indicate alterations in the dynamics of CTO in response to arterial *CO*_2_ changes in MCI patients, especially at lower frequencies (<0.05 Hz). For the CFV input (middle panel of Figure 3), the GF is similar for Controls and MCI patients at frequencies > 0.08 Hz, but considerably smaller for MCI patients at lower frequencies (by a factor of 2 on the average). Both Controls and MCI patients exhibit resonant peaks in the range 0.02–0.04 Hz. For the ABP input (right panel of Figure 3), the GF is similar for Controls and MCI patients for frequencies > 0.06 Hz, with a resonant peak in the range 0.06–0.08 Hz (the resonant peak is a bit smaller and at slightly higher frequency for MCI patients). Notably, the MCI patients exhibit higher GF values for frequencies < 0.02 Hz.

**FIGURE 4:**
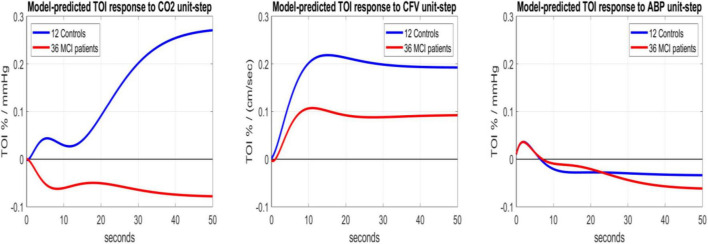
Average model-predicted TOI responses of 12 Controls (blue line) and 36 MCI patients (red line) to a unit-step input of CO_2_ (left), CFV (middle) and ABP (right), while other inputs are set to zero (baseline values).

The statistical significance of these average kernel differences between MCI patients and Controls can be examined in the context of the respective estimation variances by simulating the predictive models (defined by the respective kernels) for each unit-step input, while the other two inputs are set to zero/baseline. We can then compute model-based indices for three functional aspects of the regulation of cerebral oxygenation (determined by the respective input-output pair), as time-averages of the respective model-predicted TOI response of each MCI patients or Controls over a specified time-horizon. Figure 4 shows the average unit-step model-predicted responses over 36 MCI patients (red line) and 12 Controls (blue line) for the three different inputs (CO2, CFV, ABP). It is evident that a unit-step change of CO_2_ has the greatest effect on the TOI model-predicted response, and the effect of a unit-step ABP change is the smallest. Likewise, the difference of unit-step responses between MCI patients and Controls is greatest for the CO_2_ input and smallest for the ABP input.

According to the aforementioned logic of computing model-based indices by time-averaging of the TOI response to each of the three unit-step inputs, we can define the following indices for three specific aspects of CTO (assumed to be related to the TOI measure of relative capillary hemoglobin saturation distribution) by selecting the appropriate input-output pair and time-averaging of the TOI response over 50 s to obtain the following indices:

(1) ***Cortical CO_2_ Reactivity (CCR):*** time-average of model-predicted TOI response to unit-step CO_2_ input; Mean (SD): 0.144 (0.248) for Controls vs.–0.059 (0.244) for MCI patients, ***p = 0.022***.
(2) ***Cortical Tissue Perfusivity (CTP)****:* time-average of model-predicted TOI response to unit-step CFV input; Mean (SD): 0.184(0.187) for Controls vs. 0.085(0.138) for MCI patients, ***p = 0.115***.
(3) ***Cortical Autoregulation to Pressure (CAP):*** time-average of model-predicted TOI response to unit-step ABP input; Mean (SD):–0.022 (0.117) for Controls vs.–0.029 (0.131) for MCI patients, ***p = 0.861***.

All *p*-values are based on *t*-test of the difference of the means (with unequal variances) under the Gaussian assumption. It is evident that only the CCR index is significantly different between MCI patients and Controls (*p* < 0.05) and reverses sign on the average (from positive in Controls to negative in MCI patients). This result suggests a significant impairment of cortical CO_2_ vasomotor reactivity in MCI patients. A similar result, but with a smaller *p* = 0.006, was found in our previous work (Marmarelis et al., 2017) that did not account for the effects of CFV changes in the middle cerebral arteries (which incorporate largely the effects of possible impairment in the chemoreflex). Thus, CO_2_ vasomotor reactivity is significantly impaired in both large and small/micro cerebral vessels of MCI patients.

**FIGURE 5:**
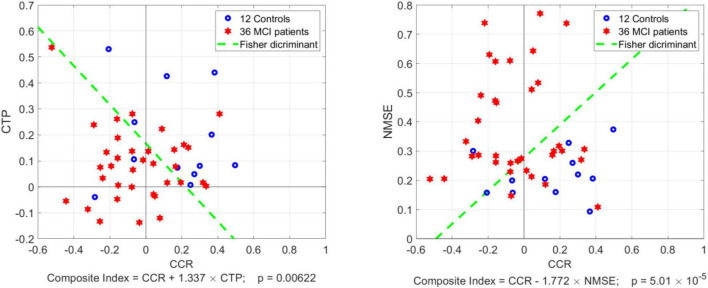
Scatter-plots of CTP vs. CCR index (left panel) and NMSE vs. CCR index (right panel) for 36 MCI patients (red) and 12 Controls (blue) with the Fisher Discriminant drawn as a dashed green line. The latter defines two Composite Indices that achieve smaller *p*-value between MCI patients vs. Controls than CCR alone: *p* = 0.006 for CCR combined with CTP, and *p* = 5 × 10^–5^ for CCR combined with NMSE.

Although the CTP index is not significantly different between Controls and MCI patients (*p* = 0.115), the mean value for MCI patients is about half the mean for Controls, suggesting the potential utility of a Composite Index defined as a linear combination of CCR and CTP according to the Fisher Discriminant (Fisher, 1936) in order to achieve a smaller *p*-value. It was found that the Composite Index: [CCR + 1.337 × CTP], which corresponds to the Fisher Discriminant, yields ***p = 0.006***. This is illustrated in the left panel of Figure 5, where the scatter-plot of CTP vs. CCR values is shown for 36 MCI patients (red) and 12 Controls (blue), along with the Fisher Discriminant drawn as a dashed green line.

We also note that the average Normalized Mean-Square Error (NMSE) of the estimated model prediction was significantly smaller in Controls vs. MCI patients: Mean (SD): 22.17 (8.06) vs. 37.95 (18.15), corresponding to ***p = 0.0001***. This suggests that there are confounding factors in MCI patients, other than the effects of the three aforementioned inputs (CO_2_, CFV, ABP), which influence the dynamic regulation of CTO and are not accounted by the employed three-input model. For example, it has been reported that the function of the neurovascular unit (Iadecola and Gorelick, 2003; Iadecola, 2004) and the molecular composition of the perivascular microenvironment (Tong et al., 2005; Girouard et al., 2010) may be altered in MCI and AD patients. These may be viewed as confounding factors that contribute to higher variability in the kernel estimates for the patients by complicating the dynamic relationships between the aforementioned inputs and the output. This in turn suggests that the NMSE of model prediction can be combined with the CCR index into a Composite Index: [CCR–1.77 × NMSE], defined by the respective Fisher Discriminant (see right panel of Figure 5), to achieve even smaller *p*-value between MCI patients and Controls (***p = 5 × 10^–5^***). Using the Fisher Discriminant (green dashed line in the right panel of Figure 5) as a “diagnostic boundary”, we can calculate the sensitivity, specificity and accuracy of the latter Composite Index in discriminating MCI patients from Controls as: 66.6, 91.6, and 72.9%, respectively.

**FIGURE 6:**
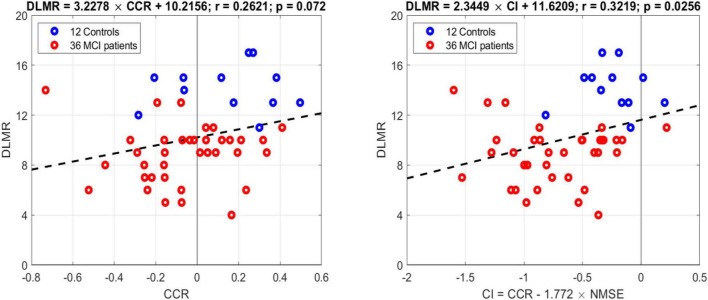
Regression lines of the DLMR scores of cognitive performance against the model-based CCR index (left panel) and the Composite index (right panel). The correlation is significant (*p* = 0.025) for the Composite index but marginal (*p* = 0.072) for the CCR index alone.

In order to examine whether these model-based indices correlate with the cognitive performance of the subjects/patients as quantified by the scores of the Delayed Logical Memory Recall (DLMR) neuropsychological test, we present in (new) Figure 6 the regression line of the DLMR scores against the model-based CCR index (left panel) and the Composite index (right panel). The correlation is significant (*p* = 0.025) for the Composite index [CCR–1.77 × NMSE] but marginal (*p* = 0.072) for the CCR index alone.

## Discussion

This paper presents the results of the application of a quantitative methodology that extracts predictive models of the dynamic regulation of cortical oxygenation (viewed as an “output” variable) as a function of concurrent spontaneous changes of blood *CO*_2_ tension and the key hemodynamic variables of arterial blood pressure and cerebral blood flow in the middle cerebral arteries (viewed as three “inputs”). These subject-specific models are used to generate model-based “physio-markers” of the regulation of cerebral perfusion in order to gain quantitative insight into the possible association between the regulation of CTO dynamics and MCI. These physio-markers cannot be evaluated for diagnostic purposes at this time, because we do not currently know what percentage of MCI patients actually have the impairment captured by these model-based indices (lack of “ground truth”). However, the potential diagnostic utility of this approach remains an important goal for future studies as more relevant knowledge is accumulated.

The employed methodology was also used recently for the quantification of *Dynamic Vasomotor Reactivity* (DVR) in the middle cerebral arteries, finding that these model-based DVR indices are significantly different between MCI patients and age-matched Controls (Marmarelis et al., 2017). This methodology was also used to examine the relation between model-based indices of heart-rate chemoreflex gain and DVR in cerebral arteries, revealing that the chemoreflex was significantly impaired in MCI patients and correlated significantly with the DVR impairment in those patients (Marmarelis et al., 2020).

The present paper utilizes the same methodological approach to examine the effects of key hemodynamic variables and blood CO_2_ tension upon CTO at the lateral prefrontal cortex (represented by the surrogate measure of *Tissue Oxygenation Index* (TOI) that is obtained *via* NIRS and actually measures the relative hemoglobin saturation distribution in the cerebral capillary arterial/venous compartments). The main findings of this study are:

(1) The response of cortical *Tissue Oxygenation Index* (TOI) to a unit-step change of blood CO_2_ (1 mmHg) was significantly different for MCI patients vs. Controls (see average TOI response traces in the left panel of Figure 4 that have opposite polarity) as quantified by the model-based *Cortical CO_2_ Reactivity* (CCR) index (*p* = 0.022), indicating impaired CTO response to transient changes of blood CO_2_ in MCI patients.

(2) The response of TOI to a unit-step change of CFV (1 cm/s) at the middle cerebral arteries was about half (on the average) in MCI patients relative to Controls (see the average blue and red TOI response traces in the middle panel of Figure 4), but the respective *Cortical Tissue Perfusivity* (CTP) index was not significantly different between MCI patients and Controls (*p* = 0.115). Nonetheless, this reduction of CTP index in MCI patients may be combined with the reduction of the CCR index in MCI patients (see #1 above) to achieve smaller *p*-value between the two groups (***p = 0.006***) *via* a Composite Index that is defined by the respective Fisher Discriminant (see left panel of Figure 5).

(3) The model prediction of TOI from spontaneous time-series data of the three inputs (CO_2_, CFV, ABP) had significantly smaller Normalized Mean-Square Error (NMSE) for Controls (***p = 0.0001***). This finding suggests that CTO is influenced in MCI patients by more factors (relative to Controls) that confound its prediction by the three inputs of the model (e.g., effects of impaired neurovascular coupling or altered perivascular microenvironment; Tong et al., 2005; Girouard et al., 2010). The NMSE of the respective model prediction can be combined with the CCR index to achieve even smaller *p*-value between MCI patients and Controls (***p = 5 × 10^–5^***) *via* a Composite Index defined by the respective Fisher Discriminant (see right panel of Figure 5).

Previous studies, including our own, have shown that the progression of cognitive impairment in patients with MCI or AD is associated with impairment of cerebral vasomotor reactivity during hypercapnia (Maeda et al., 1993; Franceschi et al., 1995; Terbor et al., 2000; de la Torre, 2002, 2004; Iadecola and Gorelick, 2003; Iadecola, 2004; Tan and Taylor, 2014; Alwatban et al., 2019). The present findings advance further our understanding of this key physiological process of cerebral flow regulation by revealing a significant differential effect of blood *CO*_2_ changes upon CTO in MCI patients relative to age-matched Controls, even after the effects of impairment of chemoreflex-mediated regulation of cerebral flow are accounted for.

An important issue that was elucidated by the Transfer Functions obtained in this study regards the frequency-response characteristics of CTO to concurrent changes of blood *CO*_2_ and key hemodynamic variables in MCI patients (see Figure 3). The magnitudes of the average Transfer Function, shown in Figure 3, are distinct between MCI patients and Controls for frequencies below 0.05 Hz, especially for the CO_2_ and CFV inputs where the MCI patients exhibit much lower gain values. This implies that if the blood CO_2_ or CFV values oscillate at frequencies below 0.05 Hz, then the induced fluctuation of CTO will be much smaller in MCI patients—possibly inducing conditions leading to the observed cognitive impairment. The physiological and clinical implications of this finding ought to be explored in future studies with regard to the mechanism that may be impaired in MCI patients and cause reduction of the CTO response to slow oscillations (< 0.05 Hz) of CO_2_ and/or CFV.

In the time-domain analysis, the average kernels shown in Figure 2 indicate distinctly different average CTO response dynamics between MCI patients and Controls in terms of the effect of transient CO_2_ changes upon the cortical TOI (see left panel of Figure 4). Notably, the average response of MCI patients was found to have opposite polarity relative to Controls. There is also considerable scaling difference between MCI patients and Controls in the average TOI response to a unit-step change in CFV (see middle panel of Figure 4), although these responses retain the same polarity and their difference does not rise to statistical significance (*p* = 0.115).

The counterintuitive result of CCR polarity reversal in MCI has some experimental corroboration in the neurovascular coupling literature (Girouard et al., 2010), where it was found that a large rise of extracellular K+ in the perivascular space adjacent to astrocytic endfeet causes a transition from vasodilation to vasoconstriction in response to astrocytic calcium waves, when this K+ rise exceeds a critical level (the K+ equilibrium potential that equals the smooth-muscle membrane potential). Thus, we can posit the hypothesis that a long-lasting alteration in the perivascular potassium (possibly caused by a “sub-clinical” chronic condition, such as chronic acidosis or inflammation) may induce reversal of polarity in cerebral CCR. This alteration may also be caused by significant increase of free radical species in the perivascular space that impede the vasodilatory action of nitric oxide, as reported in Tong et al. (2005). These hypotheses attain great importance in the context of AD pathogenesis, whereby cerebrovascular impairment or dysregulation of cerebral perfusion may prove to be an early and critical factor.

This discussion points to the confounding effects of possible dysfunction of the neurovascular unit (distinct from possible dysregulation of cerebral perfusion or oxygenation), since this would explain the increased normalized mean-square error (NMSE) in the model prediction for MCI patients after the dynamic effects of cerebral blood flow, blood *CO*_2_ and arterial blood pressure have been taken into account. In other words, these NMSE differences may pertain to changes in the cerebral microvascular environment and the neurovascular unit occurring in MCI. If this hypothesis can be validated in future studies, then this NMSE measure may offer quantitative insight into the state of the cerebral microvascular environment for diagnostic purposes.

It is useful to note that the presented methodology utilizes *canonical* linear dynamic models that are distinct from the data-based classification methods of “machine learning,” which may utilize any type of data/features in connection with *ad hoc* computational structures/models (e.g., artificial neural networks of any type among numerous alternatives) to separate two or more classes *via* iterative computational schemes. In machine learning, the validation of the results is based on comparisons between “training” and “testing” datasets—but their interpretation (if any) has to be made with reference to the employed computational structure/model and tends to be difficult due to the nonlinear elements (typically sigmoidal functions) contained therein. The results obtained *via* such machine learning methods are not unique on account of the employed iterative computational schemes (that may converge to different answers at different runs) and their dependence on the employed *ad hoc* computational structure/model. By contrast, the presented approach of linear dynamic models using the *canonical* (i.e., valid for *all* linear time-invariant dynamic input-output mappings) convolutional representation provides a unique answer for the given input-output data of each subject, which has universal validity (within the assumption of dynamic linearity) and can be interpreted readily with the well-developed concepts of Transfer Functions or Impulse Response Functions (kernels).

### Limitations of This Study

A limitation of this study is the relatively small size of the cohort that prevents proper evaluation of the model-based diagnostic physio-markers and the lack of balance in terms of the number or gender of MCI patients and control subjects in the cohort. Therefore, larger and balanced cohorts of MCI patients and Controls must be analyzed before any conclusions can be drawn regarding the potential clinical utility of the presented model-based physio-markers. In this regard, we note the confounding effects of potential vasomotor cross-sectional changes occurring in the middle cerebral arteries upon the obtained kernel estimates of subject-specific models. This is bound to increase the intragroup variability of the obtained models. Another limitation pertains to the use of *linear* dynamic models and *only two inputs*, although this system is likely to exhibit some nonlinearities and be influenced by more than two physiological “input” variables. Our group studied previously dynamic nonlinearities in this system (Marmarelis et al., 2012, 2013) and concluded that the available length of data is adequate to support reliable linear dynamic modeling of this system—but not necessarily adequate for reliable *nonlinear* dynamic modeling. The latter requires longer data-sets that may become available in the future. Nonetheless, the presented methodology is readily applicable to nonlinear dynamic modeling and may include any number of inputs and outputs (Marmarelis, 2004), provided that the requisite data are available. We note that methods used to date for cerebral oxygenation data (e.g., Al-Rawi and Kirkpatrick, 2006; Brugnara, 2021) have performed regression analysis of discrete quantities (typically averages over a certain time-interval) and do not perform dynamic analysis of time-series data of the type presented herein. Thus, there is no basis for direct comparison of the results obtained by these two distinct approaches. This underlines the novelty of the proposed approach.

## Conclusion

In conclusion, the presented approach generates model-based indices that reveal significant statistical differences of *CO*_2_ dynamic reactivity in cortical tissue combined with perfusivity from cerebral arteries to CTO in MCI patients vs. age-matched Controls (*p* = 0.006). The observed change in the dynamics of CTO in MCI patients is primarily in lower frequencies (<0.05 Hz), suggesting impairment in endocrine/metabolic (not neural) mechanisms and neurovascular coupling. These model-based indices may serve as physio-markers of possible dysregulation of CTO during transient *CO*_2_ changes in MCI patients.

## Data Availability Statement

The original contribution presented in the study are included in the article/supplementary material, further inquiries can be directed to the corresponding author/s.

## Ethics Statement

This study has been approved by the Institutional Review Board of the UT Southwestern Medical Center and Texas Health Presbyterian Hospital Dallas. The patients/participants provided their written informed consent to participate in this study.

## Author Contributions

All authors listed have made a substantial, direct, and intellectual contribution to the work, and approved it for publication.

## Conflict of Interest

The authors declare that the research was conducted in the absence of any commercial or financial relationships that could be construed as a potential conflict of interest.

## Publisher’s Note

All claims expressed in this article are solely those of the authors and do not necessarily represent those of their affiliated organizations, or those of the publisher, the editors and the reviewers. Any product that may be evaluated in this article, or claim that may be made by its manufacturer, is not guaranteed or endorsed by the publisher.

## Footnotes

1
http://adni-info.org/

## Appendix

### Outline of the Kernel Estimation Procedure From Input-Output Time-Series Data

Under the general formulation of the linear input-output model of Equation (1), the modeling task reduces to the estimation of the system kernels from input-output data. To this purpose, we employ the Laguerre expansion technique which has been shown to yield reliable kernel estimates even for noisy and relatively short datasets (Marmarelis, 2004). According to this approach, each kernel is expanded on a properly selected Laguerre basis:

(2)
$$k_{p}\left(m\right)=\sum_{j=1}^{K}C_{j}^{p}L_{j}^{p}\left(m\right)k_{x}\left(m\right)=\sum_{j=1}^{K}C_{j}^{x}L_{j}^{x}\left(m\right)k_{p}\left(m\right)=\sum_{j=1}^{K}C_{j}^{z}L_{j}^{z}\left(m\right)$$

where {*L*^i^*_*j*_(m)*} (*j = 1,…,K*) denotes the orthogonal K-dimensional Laguerre basis for the *i*-th input. These kernel expansions transform the input-output relation of Eq. (1) into Eq. (3) that involves *linearly* the unknown Laguerre expansion coefficients (which must be estimated):

(3)
$$y\left(n\right)=k_{0}+\sum_{j=1}^{K}C_{j}^{p}u_{j}\left(n\right)+\sum_{j=1}^{K}C_{j}^{x}v_{j}\left(n\right)+\sum_{j=1}^{K}C_{j}^{z}w_{j}\left(n\right)$$

where:

(4)
$$\begin{array}{c} u_{j}\left(n\right)=\sum_{m=0}^{n}L_{j}^{p}\left(n-m\right)p\left(m\right)\\ v_{j}\left(n\right)=\sum_{m=0}^{n}L_{j}^{x}\left(n-m\right)x\left(m\right)\\ w_{j}\left(n\right)=\sum_{m=0}^{n}L_{j}^{z}\left(n-m\right)z\left(m\right) \end{array}$$

Since the Laguerre expansion coefficients enter linearly in the input-output model of Eq. (3), their estimation can be achieved *via* least-squares regression (a simple and robust numerical procedure). In this study, the kernel estimation procedure utilized 4 Laguerre basis functions for each input and the Laguerre parameter alpha of 0.9 for all inputs. The selection of 4 Laguerre functions and of the alpha values was based on a search procedure that minimizes the Bayesian Information Criterion of the average model prediction over a grid of values.

## References

[B1] Ainslie P. N. Tzeng Y. C. (2010). On the regulation of the blood supply to the brain: old age concepts and new age ideas. *J. Appl. Physiol. (1985)* 108 1447–1449. 10.1152/japplphysiol.00257.2010 20299620

[B2] Ainslie P. Duffin J. (2009). Integration of cerebrovascular CO2 reactivity and chemoreflex control of breathing: mechanisms of regulation, measurement, and interpretation. *Am. J. Physiol. Regul. Integr. Comp. Physiol.* 296 R1473–R1495. 10.1152/ajpregu.91008.2008 19211719

[B3] Al-Rawi P. G. Kirkpatrick P. J. (2006). Tissue oxygen index: thresholds for cerebral ischemia using near-infrared spectroscopy. *Stroke* 37 2720–2725. 10.1161/01.STR.0000244807.99073.ae 17008623

[B4] Alwatban M. Murman D. L. Bashford G. (2019). Cerebrovascular reactivity impairment in preclinical Alzheimer’s Disease. *J. Neuroimaging* 29 493–498. 10.1111/jon.12606 30748053

[B5] Brugnara G. (2021). Dynamics of cerebral perfusion and oxygenation parameters following endovascular treatment of acute ischemic stroke. *J. Neurointerv. Surg.* 10.1136/neurintsurg-2020-017163 33762405 PMC8785045

[B6] Caldwell H. G. Howe C. A. Chalifoux C. J. Hoiland R. L. Carr J. M. J. R. Brown C. V. (2021). Arterial carbon dioxide and bicarbonate rather than pH regulate cerebral blood flow in the setting of acute experimental metabolic alkalosis. *J. Physiol.* 599 1439–1457. 10.1113/JP280682 33404065

[B7] Carter H. H. Atkinson C. L. Heinonen I. H. Haynes A. Robey E. Smith K. J. (2016). Evidence for shear stress-mediated dilation of the internal carotid artery in humans. *Hypertension* 68 1217–1224. 10.1161/HYPERTENSIONAHA.116.07698 27572152

[B8] Claassen J. A. Diaz-Arrastia R. Martin-Cook K. Levine B. D. Zhang R. (2009). Altered cerebral hemodynamics in early Alzheimer disease: a pilot study using transcranial Doppler. *J. Alzheimers Dis.* 17 621–629. 10.3233/JAD-2009-1079 19433892 PMC3210481

[B9] Cloverdale N. S. Gati J. S. Opalevych O. Perrotta A. Shoemaker J. K. (2014). Cerebral blood flow velocity underestimates cerebral blood flow during modest hypercapnia and hypocapnia. *J. Appl. Physiol.* 117 1090–1096. 10.1152/japplphysiol.00285.2014 25012027

[B10] de la Torre J. C. (2002). Alzheimer disease as a vascular disorder: nosological evidence. *Stroke* 33 1152–1162. 10.1161/01.STR.0000014421.15948.67 11935076

[B11] de la Torre J. C. (2004). Is Alzheimer’s disease a neurodegenerative or a vascular disorder? Data, dogma, and dialectics. *Lancet Neurol.* 3 184–190. 10.1016/S1474-4422(04)00683-0 14980533

[B12] Fisher R. A. (1936). The use of multiple measurements in taxonomic problems. *Ann. Eugenics* 7 179–188. 10.1111/j.1469-1809.1936.tb02137.x

[B13] Franceschi M. Alberoni M. Bressi S. Canal N. Comi G. Fazio F. (1995). Correlations between cognitive impairment, middle cerebral artery flow velocity and cortical glucose metabolism in the early phase of Alzheimer’s disease. *Dementia* 6 32–38. 10.1159/000106919 7728217

[B14] Girouard H. Bonev A. D. Hannah R. M. Meredith A. Aldrich R. W. Nelson M. T. (2010). Astrocytic endfoot Ca2+ and BK channels determine both arteriolar dilation and constriction. *Proc. Natl. Acad. Sci. U.S.A.* 107 3811–3816. 10.1073/pnas.0914722107 20133576 PMC2840528

[B15] Hachinski V. Iadecola C. (2004). Vascular cognitive impairment: introduction. *Stroke* 35:2615. 10.1161/01.STR.0000143237.11078.bc PMC3955006 20876487

[B16] Hoiland R. L. Ainslie P. N. (2016). The middle cerebral artery diameter does change during alterations in arterial blood gases and blood pressure. *J. Physiol.* 594 4073–4075. 10.1113/JP271981 27010010 PMC4806217

[B17] Iadecola C. (2004). Neurovascular regulation in the normal brain and in Alzheimer’s disease. *Nat. Rev. Neurosci.* 5 347–360. 10.1038/nrn1387 15100718

[B18] Iadecola C. (2013). Review: the pathobiology of vascular dementia. *Neuron* 80 844–866. 10.1016/j.neuron.2013.10.008 24267647 PMC3842016

[B19] Iadecola C. Gorelick P. B. (2003). Converging pathogenic mechanisms in vascular and neurodegenerative dementia. *Stroke* 34 335–337. 10.1161/01.STR.0000054050.51530.76 12574528

[B20] Iturria-Medina Y. Sotero R. C. Toussaint P. J. Mateos-Perez J. M. Evans A. C. (2016). Early role of vascular dysregulation on late-onset Alzheimer’s disease based on multifactorial data-driven analysis. *Nat. Commun.* 7:11934. 10.1038/ncomms11934 27327500 PMC4919512

[B21] Maeda H. Matsumoto M. Handa N. Hougaku H. Ogawa S. Itoh T. (1993). Reactivity of cerebral blood flow to carbon dioxide in various types of ischemic cerebrovascular disease: evaluation by transcranial Doppler method. *Stroke* 24 670–675. 10.1161/01.STR.24.5.670 8488521

[B22] Marmarelis V. Z. (2004). *Nonlinear Dynamic Modeling of Physiological Systems.* Hoboken, NJ: Wiley-Interscience. 10.1002/9780471679370

[B23] Marmarelis V. Z. Mitsis G. D. Shin D. C. Zhang R. (2016). Multiple-input nonlinear modelling of cerebral haemodynamics using spontaneous arterial blood pressure, end-tidal CO2 and heart rate measurements. *Phil. Trans. R. Soc. A* 374:20150180. 10.1098/rsta.2015.0180 27044989 PMC4822442

[B24] Marmarelis V. Z. Shin D. C. Zhang R. (2012). Linear and nonlinear modeling of cerebral flow autoregulation using Principal Dynamic Modes. *Open Biomed. Eng. J.* 6 42–55. 10.2174/1874120701206010042 22723806 PMC3377891

[B25] Marmarelis V. Z. Shin D. C. Zhang R. (2020). Dysregulation of CO2-driven heart-rate chemoreflex is related closely to impaired CO2 Dynamic Vasomotor Reactivity in MCI patients. *J. Alzheimers Dis.* 75 855–870. 10.3233/JAD-191238 32333588 PMC7369119

[B26] Marmarelis V. Z. Shin D. C. Oesterreich M. Mueller M. (2019). Quantification of dynamic cerebral autoregulation and CO2 dynamic vasomotor reactivity impairment in essential hypertension. *J. Appl. Physiol.* 128 397–409. 10.1152/japplphysiol.00620.2019 31917625 PMC7052591

[B27] Marmarelis V. Z. Shin D. C. Orme M. E. Zhang R. (2013). Model-based quantification of cerebral hemodynamics as a physiomarker for Alzheimer’s disease? *Ann. Biomed. Eng.* 41 2296–2317. 10.1007/s10439-013-0837-z 23771298 PMC3992829

[B28] Marmarelis V. Z. Shin D. C. Orme M. E. Zhang R. (2014). Model-based physiomarkers of cerebral hemodynamics in patients with mild cognitive impairment. *Med. Phys. Eng.* 36 628–637. 10.1016/j.medengphy.2014.02.025 24698010 PMC4076301

[B29] Marmarelis V. Z. Shin D. C. Tarumi T. Zhang R. (2017). Comparison of model-based indices of cerebral autoregulation and vasomotor reactivity using Transcranial Doppler versus Near-Infrared Spectroscopy in patients with amnestic Mild Cognitive Impairment. *J. Alzheimers Dis.* 56 89–105. 10.3233/JAD-161004 27911329 PMC5240580

[B30] Nicolakakis N. Hamel E. (2011). Neurovascular function in Alzheimer’s disease patients and experimental models. *J. Cereb. Blood Flow Metab.* 31 1354–1370. 10.1038/jcbfm.2011.43 21468088 PMC3130325

[B31] Petersen R. C. Doody R. Kurz A. Mohs R. C. Morris J. C. Rabins P. V. (2001). Current concepts in mild cognitive impairment. *Arch. Neurol.* 58 1985–1992. 10.1001/archneur.58.12.1985 11735772

[B32] Silvestrini M. Pasqualetti P. Baruffaldi R. Bartolini M. Handouk Y. Matteis M. (2006). Cerebrovascular reactivity and cognitive decline in patients with Alzheimer’s disease. *Stroke* 37 1010–1015. 10.1161/01.STR.0000206439.62025.97 16497984

[B33] Tan C. O. Taylor J. A. (2014). Integrative physiological and computational approaches to understand autonomic control of cerebral autoregulation. *Exp. Physiol.* 99 3–15. 10.1113/expphysiol.2013.072355 24097158 PMC3947359

[B34] Terbor C. Gora F. Weiller C. Rother J. (2000). Reduced vasomotor reactivity in cerebral micro-angiopathy: a study with near-infrared spectroscopy and transcranial Doppler sonography. *Stroke* 31 924–929. 10.1161/01.STR.31.4.924 10754000

[B35] Tong X. K. Nicolakakis N. Kocharyan A. Hamel E. (2005). Vascular remodeling versus amyloid beta-induced oxidative stress in the cerebrovascular dysfunctions associated with Alzheimer’s disease. *J. Neurosci.* 25 11165–11174. 10.1523/JNEUROSCI.4031-05.2005 16319316 PMC6725645

[B36] Verbree J. Bronzwaer A. G. T. Ghariq E. Versluis M. J. Daeman M. J. A. P. van Buchem M. A. (2014). Assessment of middle cerebral artery diameter during hypocapnia and hypercapnia in humans using ultra high-field MRI. *J. Appl. Physiol.* 117 1084–1089. 10.1152/japplphysiol.00651.2014 25190741

[B37] Willie C. K. Macleod D. B. Shaw A. D. Smith K. J. Tzeng Y. C. Eves N. D. (2012). Regional brain blood flow in man during acute changes in arterial blood gases. *J. Physiol.* 590 3261–3275. 10.1113/jphysiol.2012.228551 22495584 PMC3459041

[B38] Willie C. K. Tzeng Y. C. Fisher J. A. Ainslie P. N. (2014). Integrative regulation of human brain blood flow. *J. Physiol.* 592 841–859. 10.1113/jphysiol.2013.268953 24396059 PMC3948549

